# Direct study of the electrical properties of PC12 cells and hippocampal neurons by EFM and KPFM

**DOI:** 10.1039/c8na00202a

**Published:** 2018-11-19

**Authors:** Weidong Zhao, Wei Cui, Shujun Xu, Ling-Zhi Cheong, Deyu Wang, Cai Shen

**Affiliations:** Ningbo Institute of Materials Technology & Engineering, Chinese Academy of Sciences 1219 Zhongguan Road Ningbo Zhejiang China shencai@nimte.ac.cn; Ningbo Key Laboratory of Behavioral Neuroscience, Provincial Key Laboratory of Pathophysiology, School of Medicine, Ningbo University Ningbo Zhejiang China; School of Marine Science, Ningbo University Ningbo 315211 China

## Abstract

Electrical related properties play important roles in biological structures and functions. Herein, the capacitance gradient and local contact potential difference (CPD) of cell bodies and processes of PC12 cells (representative cells of the sympathetic nervous system), hippocampal neurons (representative cells of the central nervous system) and spines were investigated by Electrostatic Force Microscopy (EFM) and Kelvin Probe Force Microscopy (KPFM) at high lateral spatial resolution directly. The results demonstrate that the capacitance gradients of cell bodies, processes and spines of PC12 cells and hippocampal neurons are very close (in the range of 19–23 zF nm^−1^) and fit well with the theoretical calculation results (21.7 zF nm^−1^). This indicates that the differences of nerve signal activities and functions of the sympathetic and central nervous systems are not related to the electric polarization properties. The CPD of cell bodies and processes of PC12 cells is smaller than that of hippocampal neurons. The CPD of spines is much more negative than that of the cell bodies and processes. These results reveal that the surface potential is closely related to the neural signal transduction functions, and spines play vital roles in neural signal transmission. This work indicates the similarity (capacitance gradient) and differences (surface potential) of the electrical properties between the sympathetic and central nervous systems for the first time. The methods and results of this work are useful in the further study of the electrical properties in cellular activities and physiological processes.

## Abstract 2

The study of capacitance gradient by EFM (upper) and contact potential difference by KPFM (lower) of cells in lift modes.

## Graphical abstract

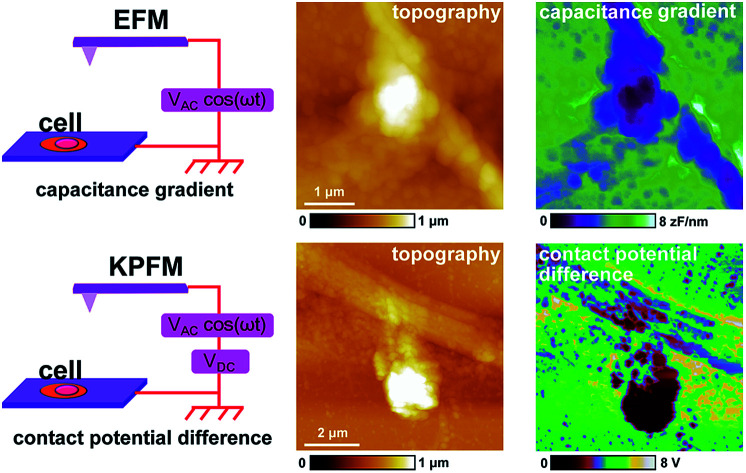

## Introduction

Dielectric and surface potential related electrical properties play important roles in various biological activities and physiological processes. Though these properties have been studied by some approaches, such as electrorotation spectroscopy,^1^ dielectric impedance spectroscopy,^2^ dielectrophoresis spectroscopy^3^ and so on, the resolution was low, or they were carried out in indirect ways. The direct investigation of these properties on cells at nanometer resolution has not been achieved yet.

The non-contact electric modes of atomic force microscopy, Electrostatic Force Microscopy (EFM) and Kelvin Probe Force Microscopy (KPFM), have been applied in imaging and quantitative analysis of various electrical properties (for instance, the capacitance gradient, dielectric constant, local charge density and surface potential) with high resolution.^4,5^ They have been used for a large variety of materials, including metals, semiconductors, organic molecules, DNA, biomembranes, cells and viruses, successfully at nanometer or even sub-nanometer resolution, which makes them appropriate approaches for studying electrical properties.^6–8^

EFM has been applied in the characterization of the electrostatic charge distributions of single walled carbon nanotubes,^9^ surface charge density of purple membranes,^10^ conductivity of thin films,^11^ lateral spreading and charge decay properties of nitride/oxide/silicon,^12^ two dimensional distributions of electrons and holes in pentacene^13^ and so on. EFM has been applied to study the electrical polarization properties of DNA, which are supposed to be important in the modulation of interactions between DNA and effector proteins.^14^ The results can be used to predict the electrostatic potential and electrostatic binding energies of DNA. EFM has been used to study the electric polarization properties of single bacteria under dry and ambient conditions.^15^ The results indicate that the effective dielectric constants of Gram-negative bacteria only increase about one fold under ambient conditions, while for Gram-positive bacteria, the effective dielectric constants increase about three fold. The internal hydration properties of endospore *Bacillus cereus* have been investigated by EFM.^16^ The dielectric constant of the endospore increased from 4 to 17 when the relative humidity changed from 0% to 80%, but only with a small increase in the height. EFM can also be applied to visualize the charge transfer in pili proteins of *Geobacter sulfurreducens* under ambient conditions with nanometer resolution. The pH and temperature dependence of charge density has also been investigated.^7^ All of this demonstrates that EFM can investigate various electrical properties of biological samples with high reliability, accuracy and reproducibility.

KPFM is a powerful tool to study the electrostatic potential and local electrochemical distributions in a large variety of materials.^5,6^ It can image the topography while measuring the surface potential or work function simultaneously at high spatial resolution and high energy sensitivity. KPFM can be applied to measure the local contact potential difference (CPD) of different surfaces of gold, platinum and other materials, and the investigations have high potential resolution (better than 0.1 mV) and spatial resolution (less than 50 nm).^5^ KPFM can also visualize the lateral dissipation and retention of injected charges in SiO_2_ and Si_3_N_4_ dielectric layers. From this, the charge diffusion coefficients, diffusion activation energy and mobility can be studied and determined.^17^ KPFM has been applied to investigate the relative direction and strength of polarization properties of ferroelectric thin films.^18,19^ Recently developed band excitation KPFM can be used in detection of force gradient and electrostatic forces.^20^ In biology, surface potential of various kinds of organic molecules, for instance organic ferroelectric oligomers, self-assembled monolayers and Langmuir–Blodgett films, has been measured by KPFM.^21–23^ The surface potential of single and double strands of DNA has also been studied by KPFM with nanometer spatial resolution and high sensitivity.^24,25^ KPFM has been applied in the label free detection of biomaterials, which is useful for the investigations of specific binding events.^25^ Besides static single biomolecules, KPFM can detect the dynamic chemical processes. The surface potential changes of optically active proteins and biomembranes induced by illumination were studied by KPFM.^26,27^ The binding behaviors of avidin-biotin and neutravidin-biotin were also detected by KPFM.^28^ KPFM has been applied in the investigations of binding behaviors of lysozyme and anti-lysozyme aptamers in a similar way. The results demonstrate that the surface potential of the protein-aptamer is lower than that of the lysozyme.^29^ Besides, KPFM has been used in the characterization of surface potential of human plasma fibrinogen and purple membranes.^30,31^ This confirms that KPFM can be applied in the detection of surface potential of any biomolecules and biomembranes that have dipole moments or charges.

All of this discussed above demonstrates that EFM and KPFM are powerful tools in the study of electrical properties of biological samples.

In this work, the capacitance gradient and CPD of PC12 cells and hippocampal neurons have been investigated by EFM and KPFM quantitatively and directly at high resolution. This will be helpful in the further investigation and understanding of the effect of electrical properties of biological materials.

## Materials and methods

### 1. Cell culture

PC12 cell lines were cultured in Dulbecco's modified Eagle medium (DMEM, Hyclone) with high glucose (4500 mg L^−1^) and 4 mM l-glutamine, containing 1% fetal bovine serum (FBS, Gibco), under 5% CO_2_ at 37 °C for 24 h, and then 1 mM cAMP (cyclic adenosine monophosphate, final concentration, Sigma) was added to induce differentiation for 24 h.

Hippocampal neurons were prepared by separating mice hippocampal tissue from newborn ICR (Institute of Cancer Research) mice as reported previously.^32–34^ The hippocampi tissues (dissected and chopped from the diencephalon) were digested by 0.25% trypsin (Invitrogen) for 15 min at 37 °C. Then the cells were dissociated and collected on coverslips. The coverslips were coated with 100 μg mL^−1^ poly-d-lysine previously. The medium was DMEM (Invitrogen) with 10% FBS (Invitrogen) and 10% F-12 (Invitrogen). The cells were cultured at 37 °C, 5% CO_2_ for 24 h. Then the medium was replaced by Neurobasal medium supplemented with 1% glutamine and 2% B27. At 5 days *in vitro*, the coverslips were added to 5 μM (final concentration) cytosine arabinofuranoside (Invitrogen) in order to reduce the growth of glial cells. Then Lipofectamine 2000 (Invitrogen) was added for the transfection of the F-GFP (farnesylated enhanced green fluorescent protein) and GFP-actin.

All the experiments were approved by the Animal care and Use Committee of the Ningbo University (Ningbo, China). Every procedure followed the instructions of the National Institutes of Health (NIH) Guide for the Care and Use of Laboratory Animals (NIH Publications no. 80-23, revised 1996).

### 2. Fluorescence microscopy

#### PC12 cells

The medium was removed and the cells were rinsed with phosphate buffered saline (PBS, 0.1 M Na_2_HPO_4_, 0.1 M KH_2_PO_4_, 0.1 M KCl and 0.1 M NaCl, pH 7.4) 3 times. Then the cells were treated with 10 μg mL^−1^ fluorescence diacetate (FDA) and were imaged by inverted fluorescence microscopy (Nikon Eclipse Ti-S). The excitation wavelength was 480 nm. The emission fluorescence (wavelength 530 nm) was collected using a 40× objective. The images were captured and processed using software NIS Elements F.

#### Hippocampal neurons

The hippocampal neurons were imaged using a laser confocal fluorescence microscope Fluoview-1000 (Olympus, Tokyo, Japan). The excitation wavelength is 488 nm. The emission fluorescence was collected using a 60× oil immersion objective at 7 days *in vitro*. The images were captured and analyzed using software Fluoview-1000 (Olympus, Tokyo, Japan).

### 3. AFM

All the AFM experiments, including ScanAsyst, EFM and KPFM modes, were performed using an AFM Icon (Bruker). All images were captured with 512 × 512 pixels.

**Fig. 1:**
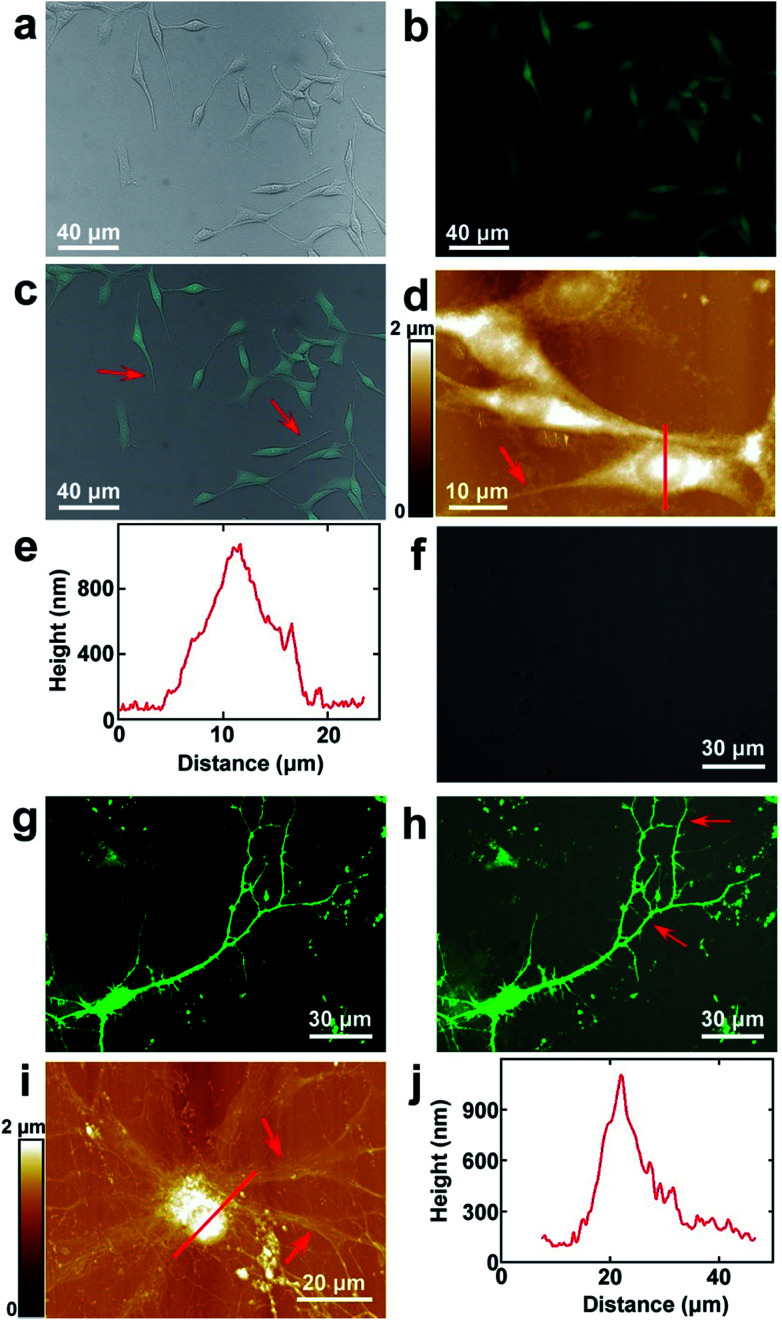
The surface morphology of PC12 cells and hippocampal neurons. (a and b) The bright field and fluorescence images of PC12 cells, respectively; (c) the merged image of (a) and (b); (d) the AFM topography of PC12 cells. The red arrows in (c) and (d) point to the processes of PC12 cells; (e) the cross sectional analysis along the red line in (d); (f and g) the bright field and fluorescence images of hippocampal neurons, respectively; (h) the merged image of (f) and (g); (i) the AFM topography of hippocampal neurons. The red arrows in (h) and (i) point to the dendrites of hippocampal neurons; (j) the cross sectional analysis along the red line in (i).

The topography images of PC12 cells and hippocampal neurons in Fig. 1 were acquired in ScanAsyst mode of AFM. In this mode, the scanning parameters can be adjusted and optimized automatically, and the samples can be scanned more gently than in the traditional typical tapping mode. The probe was SNL-10 (Bruker Corporation), and cantilever B was chosen for imaging. The top layer and back side of the cantilevers were coated with reflective Ti/Au and gold, respectively. The main specifications (nominally) are as follows: spring constant 0.12 N m^−1^; resonant frequency 23 kHz; tip radius 2 nm, which were enough to produce high quality images.

### 4. EFM and KPFM

EFM and KPFM were performed at room temperature. EFM was performed in electrical and magnetic lift mode. KPFM was performed in AM-KPFM mode. EFM and KPFM were performed by a two-pass procedure. In the first pass (main channel) the sample was scanned in tapping mode to record the topography image. Then in the second pass (interleave channel) the tip was raised to the designated lift scan height, and the amplitude shift of the cantilever with respect to the nominal amplitude shift was measured, in this way the local electrostatic properties were recorded. In order to obtain capacitance gradient images without any contribution of the topography, the lift height must be adjusted carefully as reported previously.^7^ Briefly, (i) the oscillating amplitude of the tip should be set very small in the EFM mode (about 20 mV); (ii) the height of the tip was gradually decreased until the topography can be seen (this is the pure signal of the topography); (iii) the tip was lifted 40 nm above the sample, which is more effective for a 20 nm lift height. At this lift height, the cross-talk of topography can be avoided completely.^35,36^ The CPD detection by lift mode KPFM was carried out in a similar way to avoid the influences of topography.

In the interleave channel of EFM, an alternative current (drive frequency 2 kHz, drive amplitude 4000 mV) was applied in the tip. All the topography, EFM capacitance gradient and KPFM CPD images were processed using software Nanoscope Analysis 1.80 (Bruker Corporation).

In all EFM and KPFM modes, PFTUNA (Bruker Corporation) probes were used. The triangular shaped cantilevers were made of silicon nitride. The front and back sides of the cantilevers and the tips were all coated with Pt/Ir. The coated Pt/Ir can increase the laser reflectivity and electrical conductivity of the cantilevers and tips. The main specifications of the tips are (nominal) as follows: tip radius 25 nm; resonant frequency 70 kHz; spring constant 0.4 N m^−1^.

## Results and discussion

### 1. The surface morphology of PC12 cells and hippocampal neurons

PC12 is a cell line that is derived from the pheochromocytoma of the adrenal medulla, which is usually used as the model to stimulate the functions of the sympathetic nervous system.^37^ The surface morphology images of PC12 cells acquired by fluorescence microscopy are depicted in Fig. 1a–c, which correspond to the bright field, fluorescence microscopy and merged images, respectively. As the cells were stained with FDA, they can be clearly seen from the fluorescence image (Fig. 1b), which indicated that the cultured cells possessed good enzyme activity and membrane integrity. It can be observed that the bright field and fluorescence images correspond very well (Fig. 1c). The cells were grown well on the culture dishes and composed of the cell body and processes (depicted by the red arrow in Fig. 1c). Long processes were induced by the addition of cAMP. As the resolution of fluorescence microscopy is limited, the cells were imaged by AFM to obtain higher resolution images. The topography of the cells is depicted in Fig. 1d. It indicates that the cell nucleuses show a round shape, and the size of the nucleus is 8.8 ± 0.6 μm (*N* = 10). The length of the processes varies from 10 μm to 45 μm, which demonstrates that the cells may be in different culture stages. The height of the nucleus is 1.0 ± 0.2 μm (*N* = 10), as depicted by the cross sectional analysis (Fig. 1e) along the red line in Fig. 1d.

The hippocampal neuron is a representative of the neurons of the central nervous system. The morphology of the cultured hippocampal neurons was investigated, and the bright field, fluorescence and merged images of hippocampal neurons are depicted in Fig. 1f–h, respectively. As the actin of the cells was stained with GFP and there are abundant actin in the soma and dendrites, neurons can be seen in the fluorescence image (Fig. 1g). From the merged image (Fig. 1h), it can be observed that the fluorescence signals correspond with the bright field image very well. The high resolution images were acquired by AFM and are shown in Fig. 1i. There are the soma and dendrites (pointed by red arrows). The cross sectional analysis along the red line in Fig. 1i is shown in Fig. 1j. The soma shows an elliptical shape. The sizes of the major and minor axes of the soma are 19.7 ± 1.8 μm (*N* = 10) and 14.0 ± 2.3 μm (*N* = 10), respectively. The height of the soma is 1.1 ± 0.3 μm (*N* = 10).

All of this discussed above indicates that intact and healthy PC12 cells and hippocampal neurons that include the cell body and processes are obtained and are suitable for the following studies performed by EFM and KPFM.

### 2. Investigations of the capacitance gradient of cell bodies of PC12 cells and hippocampal neurons by EFM

First, the capacitance gradient of the two cells was investigated by EFM in lift mode. In this mode, the influence of topography on the detection of electrostatic forces can be eliminated and avoided. This can be attributed to the following four reasons. First, the electrostatic forces are dominant in the lift mode. Thus the only source of the signal originates from the electrostatic force.^7^ Second, the experiments were carried out carefully to eliminate the effects of topography as described in the experimental section.^7^ Third, the accurate detection of capacitance gradient can only be affected by the abrupt changes in topography. In this work, this will only happen near the edges of cells. The majority of the cell surface will not be affected.^38,39^ Fourth, it requires a long time to obtain capacitance gradient images (about 1 h). Severe cross-talk between the surface topography and the capacitance gradient can only be generated at high imaging speed.^40^ Thus capacitance gradient images without the cross-talk of topography can be obtained in this lift mode EFM approach. Pure CPD signal images without the topography cross-talk can also be acquired by lift mode KPFM following a similar manner.

**Fig. 2:**
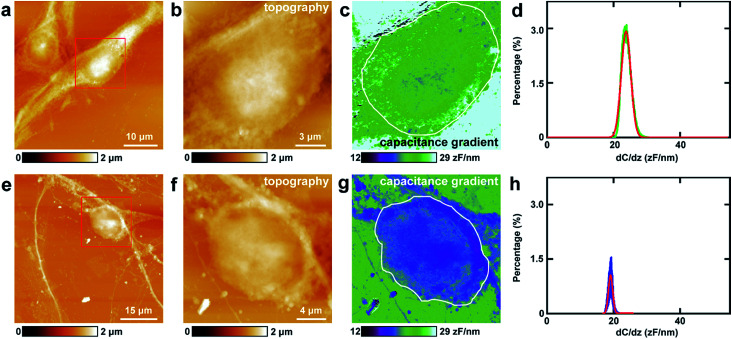
The capacitance gradient analysis of the cell bodies of PC12 cells and hippocampal neurons. (a) The topography of PC12 cells; (b) the magnified image of the red square area in (a); (c) the corresponding capacitance gradient image; (d) the histogram of distributions of capacitance gradient of the area circled with a white line in (c); (e) the topography of hippocampal neurons; (f) the magnified image of the red square area in (e); (g) the corresponding capacitance gradient image; (h) the histogram of distributions of capacitance gradient of the area circled with a white line in (g).

This approach was first utilized to investigate the capacitance gradient of the cell body of PC12 cells. The topography of PC12 cells is shown in Fig. 2a, which shows intact and well grown cells. The magnified topography image of the cell body is shown in Fig. 2b, and Fig. 2c is the corresponding capacitance gradient image. The electrostatic forces were measured at the sub-picoNewton level.^41^ The corresponding histogram of distribution of capacitance gradient is shown in Fig. 2d. From the fitted normal distribution, it can be calculated that the mathematical expectation, standard deviation and FWHM (full width at half maximum) are 23.7 ± 0.0 zF nm^−1^, 1.4 zF nm^−1^ and 3.3 zF nm^−1^, respectively. It can be seen that there is no direct relationship between the capacitance gradient and the surface height, which further confirms that the capacitance gradient values obtained in the lift mode are not affected by the topography.

Then the same methods were applied to investigate the capacitance gradient of the soma of hippocampal neurons. The topography of neurons is depicted in Fig. 2e, and the magnified topography of the soma (red square area in Fig. 2e) is shown in Fig. 2f. Fig. 2g is the corresponding capacitance gradient image of Fig. 2f. The corresponding histogram of distribution of capacitance gradient is depicted in Fig. 2h. From the fitted normal distributions, it can be calculated that the mathematical expectation, standard deviation and FWHM are 19.1 ± 0.0 zF nm^−1^, 0.6 zF nm^−1^ and 1.5 zF nm^−1^, respectively. The mathematical expectation is lower than that in PC12 cells by 4.6 zF nm^−1^, which means that the averaged value of capacitance gradient of hippocampal neurons is less than that of PC12 cells. This difference in capacitance gradient can also be seen from the images (Fig. 2c and g) directly. From the definition of capacitance, it can be concluded that the cell body of neurons can hold less charge than that of PC12 cells. The standard deviation and FWHM of the distribution of capacitance gradient in hippocampal neurons are less than those in PC12 cells, which demonstrates that the capacitance gradient distribution is more concentrated in hippocampal neurons. From the capacitance gradient measurements, the related dielectric properties, such as dielectric constant, can be estimated and compared. As capacitance gradient of cell bodies of the two types of cells are very close, it may be deduced that the corresponding dielectric constants of the cell bodies of the two types of cells are also very close.

There is another method to estimate the capacitance gradient. For the sphere-plate capacitor, the capacitance gradient between the tip and the sample can be given by eqn (1):

1 *C*′ = 2π*ε*_0_*R*^2^/*z*^2^

where *C*′ is the capacitance gradient, *ε*_0_ is the vacuum permittivity, *R* is the tip radius (in this experiment, 25 nm), and *z* is the lift distance (in this experiment, 40 nm).^42^ Thus it can be calculated that the capacitance gradient is 21.7 zF nm^−1^, which is well consistent with the experimental results.

Thus the capacitance gradient of cell bodies of PC12 cells and hippocampal neurons was investigated at high spatial (nanometer) and electrical resolution (0.1 zF nm^−1^) *in situ* directly.

### 3. Investigations of the capacitance gradient of processes of PC12 cells and hippocampal neurons by EFM

**Fig. 3:**
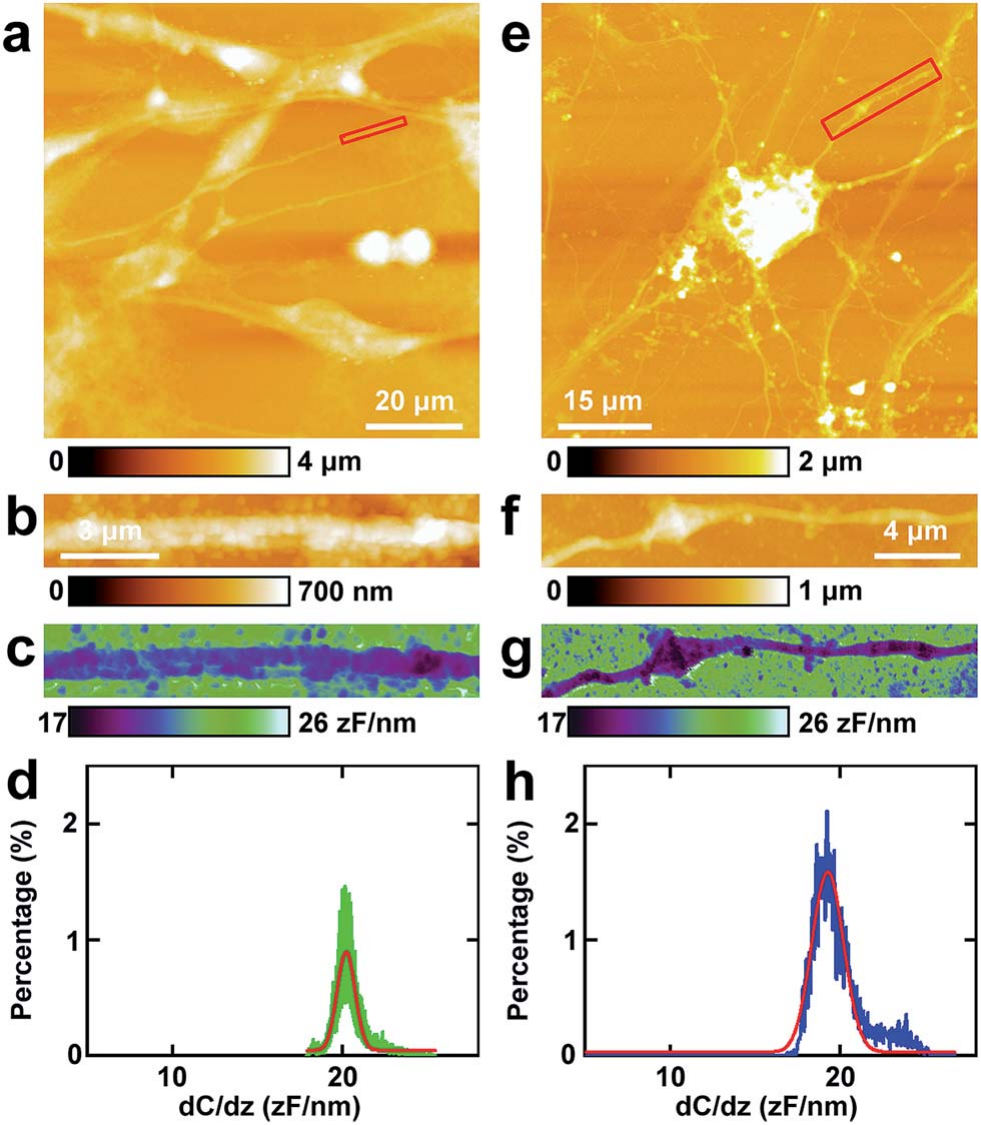
The capacitance gradient analysis of the processes of PC12 cells and hippocampal neurons. (a) The topography of PC12 cells; (b) the magnified image of the red square area in (a); (c) the corresponding capacitance gradient image; (d) the histogram of capacitance gradient distribution in (c); (e) the topography of hippocampal neurons; (f) the magnified image of the red square area in (e); (g) the corresponding capacitance gradient image; (h) the histogram of capacitance gradient distribution in (g).

Then similar EFM experiments were carried out in the processes of PC12 cells and hippocampal neurons. The results are depicted in Fig. 3.

The topography of PC12 cells is shown in Fig. 3a. Then a process was chosen and the topography image is depicted in Fig. 3b, and the corresponding capacitance gradient image is depicted in Fig. 3c. From the distribution histogram of the capacitance gradient (Fig. 3d), it can be seen that the majority capacitance gradient of the processes of the PC12 cells is at 20.2 ± 0.1 zF nm^−1^, and the standard deviation and FWHM of the fitted normal distribution are 0.5 zF nm^−1^ and 1.2 zF nm^−1^, respectively.

The morphology of neurons is depicted in Fig. 3e. The magnified topography image of the process (dendrite) is shown in Fig. 3f, and the corresponding capacitance gradient is shown in Fig. 3g. The distribution of capacitance gradient of dendrites in nerve cells is centered at 19.3 ± 0.1 zF nm^−1^ (Fig. 3h), and the standard deviation and FWHM of the fitted normal distribution are 0.9 zF nm^−1^ and 2.2 zF nm^−1^, respectively. The capacitance gradient values of processes of the two cells also correspond with the theoretical calculations (eqn (1)) very well. In the distribution histograms of capacitance gradient of processes of PC12 cells and nerve cells, there is only one major peak. The values of the distribution peaks of capacitance gradient of processes of PC12 cells and nerve cells are very close. The capacitance gradient distribution of processes of PC12 cells is more concentrated than that of neurons. From the capacitance gradient measurements, the related dielectric properties, such as dielectric constant, can be estimated and compared. As the capacitance gradients of processes of the two types of cells are very close, it may be deduced that the corresponding dielectric constants of the processes of the two types of cells are also very close. This may demonstrate that the differences of nerve signal activities and functions of the sympathetic and central nervous systems are not due to the electric polarization properties (represented by the capacitance gradient and related dielectric constant).

### 4. Investigations of the CPD of cell bodies of PC12 cells and hippocampal neurons by KPFM

**Fig. 4:**
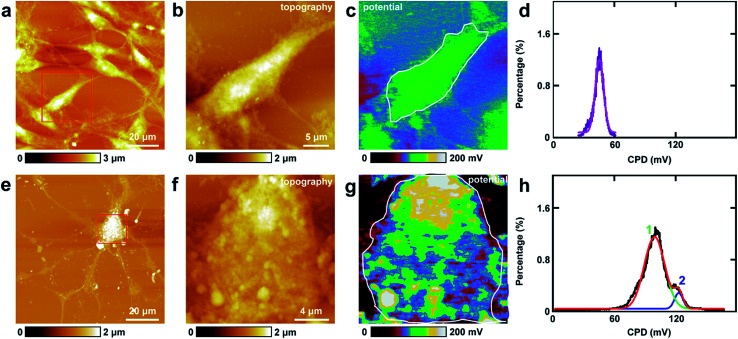
The detection of CPD of cell bodies of PC12 cells and hippocampal neurons. (a) The topography of PC12 cells; (b) the magnified image of the red square area in (a); (c) the corresponding CPD image; (d) the histogram of the CPD distribution of the cell body (area circled with a white line in (c)); (e) the topography of hippocampal neurons; (f) the magnified image of the red square area in (e); (g) the corresponding CPD image; (h) the histograms of the CPD distributions of the cell body (area circled with a white line in (g)).

The study of CPD of PC12 cells and neurons was performed by lift mode KPFM. First, the cell bodies of the two cells were investigated. The topography images of PC12 cells and neurons are displayed in Fig. 4a and e, respectively. Then the cell bodies of the two types of cells were imaged at high magnification (Fig. 4b and f). The CPD images are depicted in Fig. 4c and g, respectively. It can be observed that in the cell body of PC12 cells, the CPD has an almost homogeneous distribution (Fig. 4d). The maximum distribution is at 45.1 ± 0.1 mV, and the standard deviation and FWHM are 7.0 mV and 16.4 mV, respectively. On the other hand, for the neurons, the situation is quite different as depicted in Fig. 4h. The CPD is heterogeneously distributed in the cell body. There are two peaks in the histogram of distribution of CPD (Fig. 4h), which are at 104.1 ± 0.3 mV (main peak) and 123.8 ± 0.9 mV (shoulder peak). As *V*_CPD_ = *V*_sample_ − *V*_tip_, and in all the KPFM experiments the tip and scanning parameters are the same, it can be supposed that *V*_tip_ values are the same in all KPFM experiments. Thus from the results, it can be concluded that the surface potential difference between the two samples equals the CPD difference of the two samples. Therefore the surface potential of cell bodies of neurons is larger than that of PC12 cells by 59 mV.

The measurements of CPD by KPFM were performed in air and dried cells. From the previous study, it can be concluded that the CPD of dried biomaterials (such as protein/DNA nanoarray) is close to the values that were obtained in liquid by other approaches.^28,43^ Thus the CPD measured by KPFM under dry conditions can reflect some aspects of the electrical properties of physiological conditions of cells.

### 5. Investigations of the CPD of processes of PC12 cells and hippocampal neurons by KPFM

**Fig. 5:**
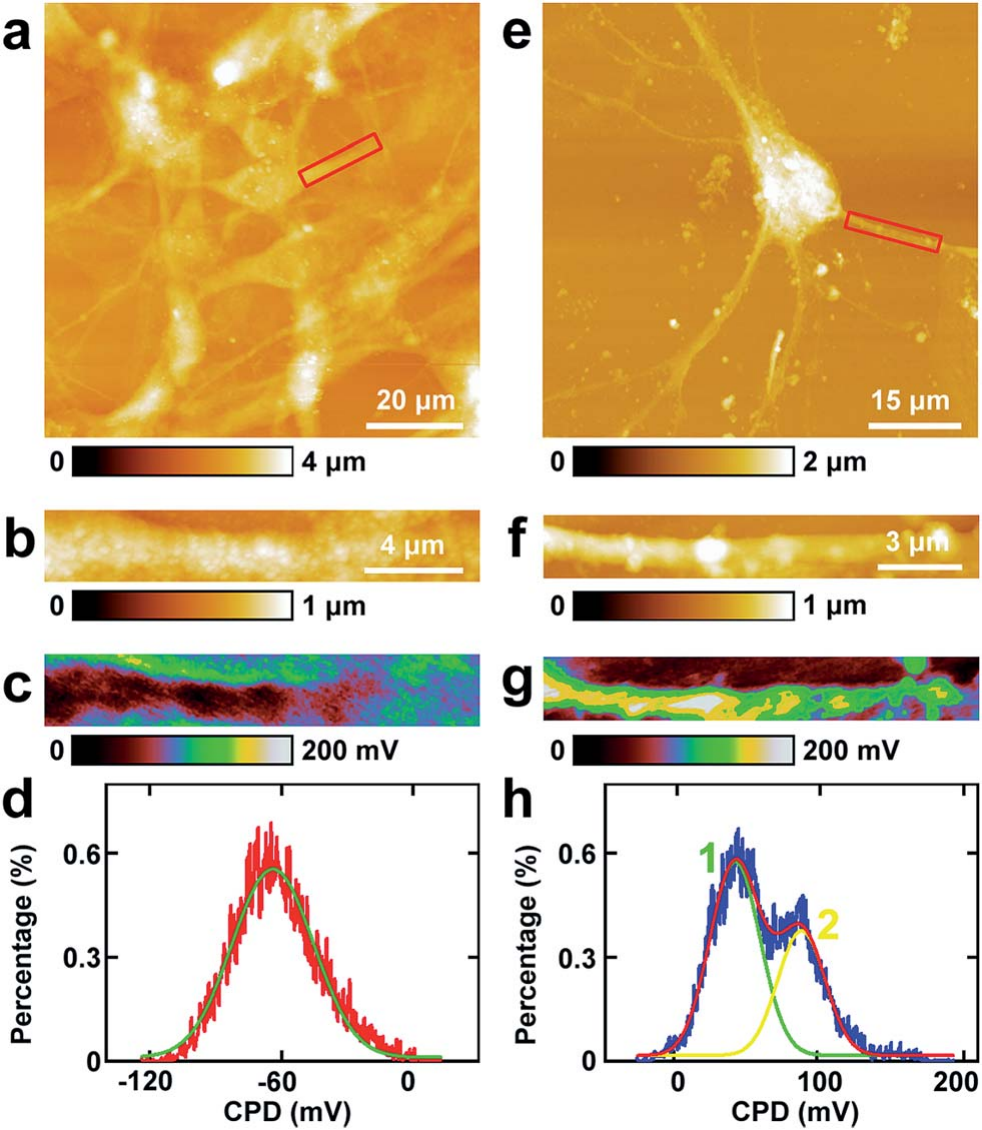
The detection of CPD of processes of PC12 cells and hippocampal neurons. (a) The topography of PC12 cells; (b) the magnified image of the red square area in (a); (c) the corresponding CPD image; (d) the histogram of the CPD distribution in (c); (e) the topography of hippocampal neurons; (f) the magnified image of the red square area in (e); (g) the corresponding CPD image; (h) the histogram of the CPD distribution in (g).

Similar KPFM experiments were performed in the processes of PC12 cells and hippocampal neurons, and the results are depicted in Fig. 5. The intact PC12 cells are shown in Fig. 5a and the cells grew very well on the Petri dish. Then a typical process of the cells was chosen and is depicted in Fig. 5b. From the CPD image (Fig. 5c) and the corresponding distribution histogram of the CPD (Fig. 5d), it can be observed that the CPD shows a rather uniform distribution. The peak of the surface distribution is at −64.1 ± 0.2 mV, and the standard deviation and FWHM are 18.5 mV and 43.6 mV, respectively.

From the topography image of hippocampal neurons (Fig. 5e), a typical dendrite was chosen and the topography image is depicted in Fig. 5f. Fig. 5g is the corresponding CPD image of Fig. 5f. It can be concluded that the CPD of dendrites shows heterogeneous distribution (Fig. 5g) and can be separated into two distinct areas, the green and the yellow area in Fig. 5g. As depicted in the histogram in Fig. 5h, there are two peaks in the fitted distribution. The main peak is at 42.8 ± 0.5 mV, which corresponds with the green area in Fig. 5g. The shoulder peak is at 89.0 ± 0.8 mV, which corresponds with the yellow area in Fig. 5g. The surface potential of processes (dendrites) in neurons is greater than that in PC12 cells by 106.9 mV, which may decrease the intake of Na^+^ and increase the exclusion of K^+^. For the processes of PC12 cells, the effect may be opposite in comparison with dendrites of neurons.

### 6. Investigations of the electrical properties of spines in hippocampal neurons

**Fig. 6:**
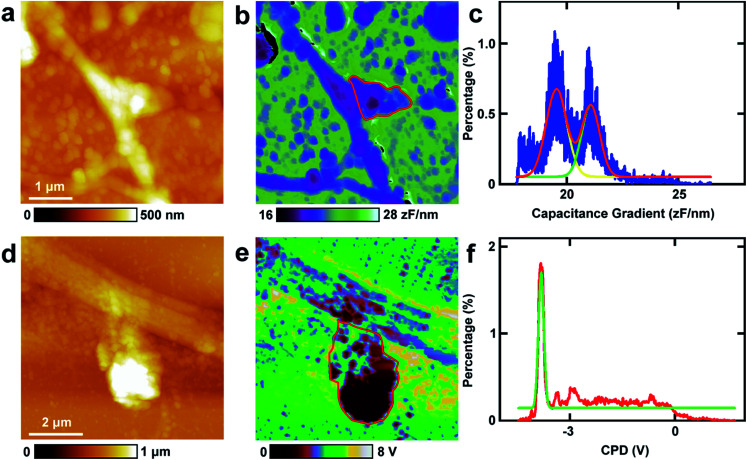
The investigations of capacitance gradient and CPD of spines of neurons. (a) The topography of a spine in a neuron; (b) the corresponding capacitance gradient image of (a); (c) the histogram of the capacitance gradient distribution in spines as circled with a red line in (b); (d) the topography of a spine in a neuron; (e) the corresponding CPD image of (d); (f) the histogram of the CPD distribution in spines as circled with a red line in (e).

The spines in the processes of neurons, which can grow up and develop as synapses, play vital roles in nerve signal propagation.^44^ But there are few studies on the electrical properties of spines. Here EFM and KPFM were applied to investigations of the related electrical properties of spines at nanometer resolution directly. The results are displayed in Fig. 6. The topography images of the typical spines in neurons are shown in Fig. 6a and d. The spines show different shapes, and the sizes of the spines are about 1 μm, which is in accordance with the previous results.^45,46^ Then the capacitance gradient of the spine was investigated by EFM and the result is depicted in Fig. 6b. The histogram of the distribution of the capacitance gradient of the spine is shown in Fig. 6c. There are two peaks, which are at 19.5 ± 0.0 zF nm^−1^ (main peak) and 21.1 ± 0.0 zF nm^−1^ (shoulder peak), respectively. The two peaks occupy almost the same areas of the spine, which corresponds with the results from eqn (1) very well. These data are very close to those of processes of PC12 cells and neurons, which further demonstrates that the capacitance and dielectric properties do not play vital roles in the differences of nerve signal activities and functions of the sympathetic and central nervous systems.

Then KPFM was performed in the spine to measure the CPD. The corresponding CPD image is displayed in Fig. 6e. The distribution histogram of the CPD is depicted in Fig. 6f. The major peak is at −3.8 ± 0.0 V. The standard deviation and FWHM of the fitted normal distribution are very small (0.1 mV and 0.2 mV, respectively), which demonstrates the distribution of CPD of the spine is very narrow. As in this work all the scanning parameters were the same in the KPFM experiments (this means that *V*_tip_ is the same), it can be concluded that the surface potential of spines are much negative than that of soma and dendrites of neurons and may be in closely with the nerve signal transduction. This may also be closely related to the electron dense substrates in postsynaptic density (PSD). These electron dense substrates can only exist in the PSD of spines, and thus the CPD of spines is more negative than that in cell bodies and processes. This negative potential may increase the activity of cations and decrease the activity of anions. Among various types of cations, Ca^2+^ plays important roles in the release of neurotransmitters. The high negative CPD of spines may increase the activity of Ca^2+^ and thus stimulate the release of neurotransmitters and increase the activity of spines.

## Conclusions

In this work, the capacitance gradient and CPD of cell bodies and processes of PC12 cells, hippocampal neurons and spines were investigated at nanometer lateral resolution directly (summarized in Table 1). The capacitance gradients of the cell bodies and processes of PC12 cells, hippocampal neurons and spines are very close (between 19 and 23 zF nm^−1^), which indicates that the differences of nerve signal activities and functions of the sympathetic and central nervous systems are related to the electric polarization properties. The CPD of cell bodies and processes of PC12 cells is lower than that of neurons. For the spines of the dendrites in the neurons, the CPD is much more negative than that of cell bodies and processes of neurons. The signal transduction and propagation functions may contribute to this high negative CPD. As the generation and propagation of nerve signals are in the form of ion flow, they can be affected by the surface potential directly. The differences of CPD between the two cells may indicate the cause of the differences of nerve signal activities. The nerve activities may be regulated by the adjustment of surface potential (especially surface potential of spines).

**Table 1:** Summary of the capacitance gradient and CPD of PC12 cells and hippocampal neuron

		Capacitance gradient (zF nm^−1^)	CPD (mV)
PC12	Cell body	23.7	45.1
	Process	20.2	−64.1
Hippocampal neuron	Cell body	19.1	104.1
	Process	19.3	42.8
	Spine	19.5	−3.8 × 10^3^

In summary, for the first time the similarity and difference of electrical properties of the sympathetic and central nervous systems were studied, which provide new insights into the understanding of the functions and mechanisms of the two types of nervous systems. The methods and conclusions of our work may be useful for further study of roles of electrical properties in nervous activity and other biological activities.

## Conflicts of interest

There are no conflicts to declare.

## Supplementary Material
